# Factors associated with hyponatremia in patients with acute spinal cord injury: a systematic review and meta-analysis

**DOI:** 10.3389/fmed.2026.1829242

**Published:** 2026-05-05

**Authors:** Huan Li, Dan Qiao, Kehui Hu

**Affiliations:** 1 North Sichuan Medical College, Nanchong, China; 2 Suining Central Hospital, Suining, China

**Keywords:** acute spinal cord injury, hyponatremia, meta-analysis, risk factors, systematic review

## Abstract

**Background:**

Hyponatremia is a prevalent electrolyte disorder in patients with acute spinal cord injury (SCI), which can significantly increase the risk of adverse complications and impair long-term prognosis. Despite its clinical significance, the existing evidence on the factors associated with hyponatremia in this patient population remains fragmented and inconsistent, leading to uncertainties in clinical prevention and management.

**Methods:**

Systematic searches were conducted in PubMed, Embase, Web of Science, Cochrane Library, and Chinese databases including CNKI, Wan fang, and VIP, retrieving relevant studies from the inception of each database up to December 20, 2025. Included observational studies reporting factors associated with hyponatremia in patients with acute spinal cord injury. Two researchers independently performed literature screening, data extraction, and quality assessment. Results were synthesized using a random-effects model to calculate odds ratios (OR) with 95% confidence intervals (CI), with assessments of heterogeneity, publication bias, and sensitivity analyses.

**Results:**

Twelve eligible studies involving 2,355 patients were included in the meta-analysis. The results indicate an association between upper cervical spine injury [OR = 6.05, 95%CI (2.79, 13.10)], complete SCI [OR = 5.35, 95%CI (3.15, 9.07)], hypoproteinemia [OR = 2.96, 95%CI (1.10, 7.92)], infection [OR = 2.20, 95%CI (1.67, 2.90)], and hyponatremia in patients with SCI.

**Conclusion:**

This meta-analysis demonstrates that hyponatremia in patients with acute SCI is closely related to the level and severity of spinal cord injury, with upper cervical spine injury and complete SCI being potential major risk indicators. Additionally, hypoproteinemia and infection are important associated factors. These findings highlight the need for targeted monitoring and intervention of high-risk patients to reduce the incidence of hyponatremia and improve clinical outcomes.

## Background

Acute spinal cord injury (SCI) is a severe traumatic disorder of the central nervous system, often caused by traumatic factors such as traffic accidents, falls from heights, and sports injuries (1). It is characterized by sudden onset, high disability rates, and significant socioeconomic burden. With improvements in emergency care and intensive care, early survival rates have markedly improved (2). However, secondary complications remain critical factors affecting prognosis, with electrolyte disturbances being particularly common (3). Hyponatremia, one of the most prevalent water-electrolyte abnormalities, occurs frequently in patients with SCI (4). Its clinical manifestations range from mild fatigue and confusion to severe convulsions and cerebral edema, potentially becoming life-threatening (5, 6). Consequently, it receives significant clinical attention.

The pathogenesis of hyponatremia following SCI is complex, typically attributed to multifactorial mechanisms including autonomic dysfunction, syndrome of inappropriate antidiuretic hormone secretion (SIADH), and cerebral salt-wasting syndrome (7). SCI, particularly high-level injuries, may disrupt neurohumoral regulatory pathways, leading to water-sodium metabolism imbalance. Concurrent inflammatory responses, stress states, and clinical interventions such as massive fluid resuscitation or diuretic use may further elevate hyponatremia risk. Additionally, injury severity, mechanical ventilation support, and concurrent infections are considered potential contributing factors (8, 9). However, existing studies present inconsistent conclusions regarding relevant associated factors. Differences in study populations, diagnostic criteria, and statistical methods among research reports contribute to a degree of fragmentation and uncertainty in the clinical evidence (10).

Identifying factors associated with hyponatremia holds significant clinical importance. Early recognition of high-risk patients not only facilitates enhanced monitoring and preventive interventions but may also reduce severe complications, shorten hospital stays, and improve functional recovery outcomes (11). Nevertheless, most existing studies on factors associated with hyponatremia in patients with SCI are single-center observational studies with limited sample sizes and insufficient statistical power, making it challenging to generate high-quality, comprehensive evidence (12). Furthermore, there is a lack of systematic integrated research that quantitatively assesses and compares different risk factors, thereby limiting the application of evidence-based medicine in clinical practice.

Therefore, this study conducted a systematic review and meta-analysis based on existing literature to comprehensively evaluate factors associated with hyponatremia in patients with SCI. The aim is to provide evidence-based guidance for clinical risk stratification management and early intervention strategy development, while offering reference directions for future high-quality research.

## Methods

This systematic review and meta-analysis was conducted in accordance with the PRISMA guidelines (13). It was registered in PROSPERO under registration number CRD420261303758.

### Literature retrieval

A systematic search was conducted in PubMed, Embase, Web of Science, the Cochrane Library, CNKI, Wan fang, and VIP from inception to December 20, 2025. The search strategy combined subject headings and free-text terms for three main concepts: acute spinal cord injury, hyponatremia, and associated factors/predictors. Boolean operators were used to combine these concepts, and the strategy was adapted for each database. Reference lists of included studies were also manually screened. The full search strategies for all databases are presented in Supplementary Table S1.

### Inclusion and exclusion criteria

Inclusion Criteria: (1) Study subjects were patients with acute spinal cord injury; (2) The study reported the incidence of hyponatremia and analyzed associated risk factors; (3) Study design was a cohort study or case–control study; (4) Provided extractable or calculable effect size data; (5) Full text was available.

Exclusion Criteria: (1) Reviews, case reports, conference abstracts, or animal studies; (2) Subjects not meeting the definition of acute spinal cord injury; (3) Failure to report risk factor analysis or inaccessible data; (4) Duplicate publications or studies with overlapping data.

### Literature screening and data extraction

Two researchers independently screened the literature. Initial screening was performed by reviewing titles and abstracts, followed by full-text reading to determine final inclusion. Disagreements were resolved through discussion or third-party adjudication. Standardized data extraction forms were used to collect information including first author, publication year, study region, study design, sample size, patient characteristics, hyponatremia diagnostic criteria, and effect sizes of relevant associated factors.

### Quality evaluation

The risk of bias in the included studies was evaluated independently by two investigators, and the results will be cross-checked. For cohort and case–control studies, the Newcastle Ottawa Scale (NOS (14)) will be used to assess quality. The NOS evaluates studies based on three dimensions: population selection, comparability, and exposure or outcome, with eight items totaling nine points. Scores range from 0 to 4 (low quality), 5 to 6 (moderate quality), and 7 to 9 (high quality). Studies scoring 0–4 will be excluded.

### Statistical analysis

All statistical analyses were performed using Stata 15.0 software. For risk factor analysis, odds ratios (ORs) and their 95% confidence intervals (CIs) were extracted as effect size measures. When both crude/univariable and multivariable-adjusted ORs were reported, the multivariable-adjusted ORs were preferentially extracted. If multiple adjusted models were available, the estimate from the most fully adjusted model was selected. Crude estimates were used only when adjusted estimates were not reported. Given potential clinical and methodological differences among included studies in terms of study design, population characteristics, and hyponatremia diagnostic criteria, all meta-analyses employed random-effects models. Model parameters utilized the DerSimonian–Laird method to calculate between-study variance (τ^2^) for more robust and conservative effect estimates.

Inter-study heterogeneity was assessed using the Cochran *Q* test and *I*^2^ statistic, where *I*^2^ values represent low (<25%), moderate (25–50%), and high heterogeneity (>50%), respectively. When significant heterogeneity was present, sensitivity analyses were conducted by sequentially excluding individual studies (leave-one-out analysis) to evaluate result robustness. Concurrently, the consistency of results obtained from different effect model parameter estimation methods was compared to validate the reliability of conclusions. Publication bias was visually assessed using funnel plots and statistically validated via Egger’s linear regression test and Begg’s rank correlation test; when potential bias was detected, further correction was performed using trim-and-fill methods. All statistical tests were two-sided, with *p* < 0.05 indicating statistical significance.

## Results

### Literature search and screening results

**Figure 1:**
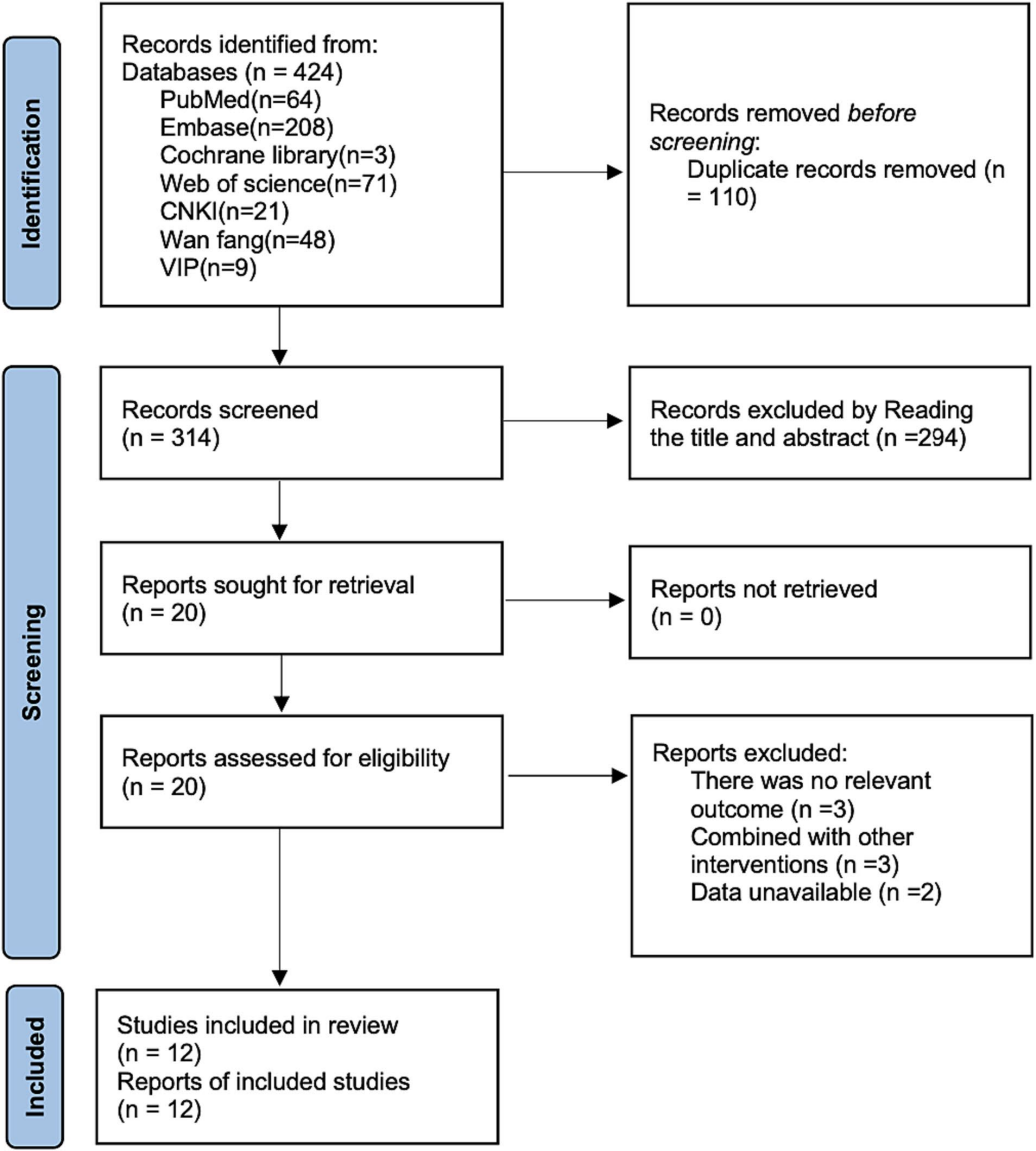
Literature search flow chart.

Searches of PubMed (*n* = 64), Embase (*n* = 208), Cochrane Library (*n* = 3), Web of Science (*n* = 71), CNKI (*n* = 21), Wan fang (*n* = 48), and VIP (*n* = 9), a total of 424 articles were identified. After removing 110 duplicate publications, 294 articles were excluded based on title and abstract review, and 8 articles were excluded after full-text review. Ultimately, 12 articles (15–26) were included. The detailed search flowchart is shown in Figure 1.

### Basic characteristics of included study

A total of 12 eligible studies involving 2,355 patients with acute SCI were included, among whom 1,144 developed hyponatremia. The studies spanned publication dates from 2012 to 2025, with research locations primarily concentrated in China, supplemented by one study each from Japan and Thailand. The predominant study design was cohort studies (10 studies), supplemented by 2 case–control studies. The mean age reported across included studies ranged from 40.2 to 62.1 years. All studies employed consistent hyponatremia diagnostic criteria—serum sodium concentration below 135 mmol/L—ensuring comparability of outcome definitions. Specific baseline characteristics are detailed in Table 1.

**Table 1:** Basic characteristics of the included studies.

Study	Year	Country	Study design	Sample size	Number of hyponatremia	Gender (M/F)	Mean age (years)	Diagnostic criteria for hyponatremia
Chavasiri	2022	Thailand	Cohort study	123	54	98/25	47.2	Serum sodium concentration <135 mmol/L
Li	2025	China	Cohort study	317	110	266/51	48	Serum sodium concentration <135 mmol/L
Nakao	2012	Japan	Case control	172	86	149/23	62.1	Serum sodium concentration <135 mmol/L
Song	2018	China	Cohort study	270	82	214/56	45	Serum sodium concentration <135 mmol/L
JC Yan	2016	China	Cohort study	369	148	226/143	45.3	Serum sodium concentration <135 mmol/L
CC Feng	2017	China	Cohort study	210	115	176/34	51.6	Serum sodium concentration <135 mmol/L
SH Tang	2012	China	Case control	62	31	37/25	40.2	Serum sodium concentration <135 mmol/L
YL Jing	2023	China	cohort study	230	128	192/38	54.4	Serum sodium concentration <135 mmol/L
X Wang	2020	China	cohort study	86	42	45/41	60	Serum sodium concentration <135 mmol/L
MM Luo	2024	China	cohort study	93	54	71/22	40.2	Serum sodium concentration <135 mmol/L
F Zheng	2017	China	cohort study	302	241	178/124	43.34	Serum sodium concentration <135 mmol/L
J Bao	2013	China	cohort study	121	53	100/21	49.2	Serum sodium concentration <135 mmol/L

### Risk of bias results

The methodological quality of the included studies was assessed using the Newcastle–Ottawa Scale. Among the 10 cohort studies, scores ranged from 6 to 9, including 3 studies with 9 points, 2 with 8 points, 2 with 7 points, and 3 with 6 points. According to our predefined criteria, studies scoring 7–9 were considered high quality, whereas those scoring 5–6 were considered moderate quality. Therefore, the cohort studies were of moderate to high methodological quality overall. The two case–control studies scored 9 and 8 points, respectively, and were both classified as high quality. Detailed quality assessment results are presented in Table 2.

**Table 2:** NOS scores results.

Cohort study									
Study	Representativeness of the exposed group	Selection of non-exposed groups	Determination of exposure factors	Identification of outcome indicators not yet to be observed at study entry	Comparability of exposed and unexposed groups considered in design and statistical analysis	design and statistical analysis	Adequacy of the study’s evaluation of the outcome	Adequacy of follow-up in exposed and unexposed groups	Total scores
Chavasiri2022	*	*	*	*	**	*	*	*	9
Li2025	*	*	*	/	**	*	*	*	8
Song2018	*	*	*	/	**	*	*	*	8
JC Yan2016	*	*	*	*	**	*	*	*	9
CC Feng2017	*	*	*	*	**	*	*	*	9
YL Jing2023	*	*	*	/	*	*	*	*	7
X Wang2020	*	*	*	/	/	*	*	*	6
MM Luo2024	*	*	*	/	*	*	*	*	7
F Zheng2017	*	*	*	/	/	*	*	*	6
J Bao2013	*	*	*	/	/	*	*	*	6

Case control									
Study	Is the case definition adequate?	Representativeness of the cases	Determination of control group	Definition of Controls	Comparability of cases and controls based on the design or analysis	Ascertainment of exposure	Same method of ascertainment for cases and controls	Non response	Total scores
Nakao2012	*	*	*	*	**	*	*	*	9
SH Tang 2012	*	*	*	/	**	*	*	*	8

### Upper cervical spine injury

**Figure 2:**
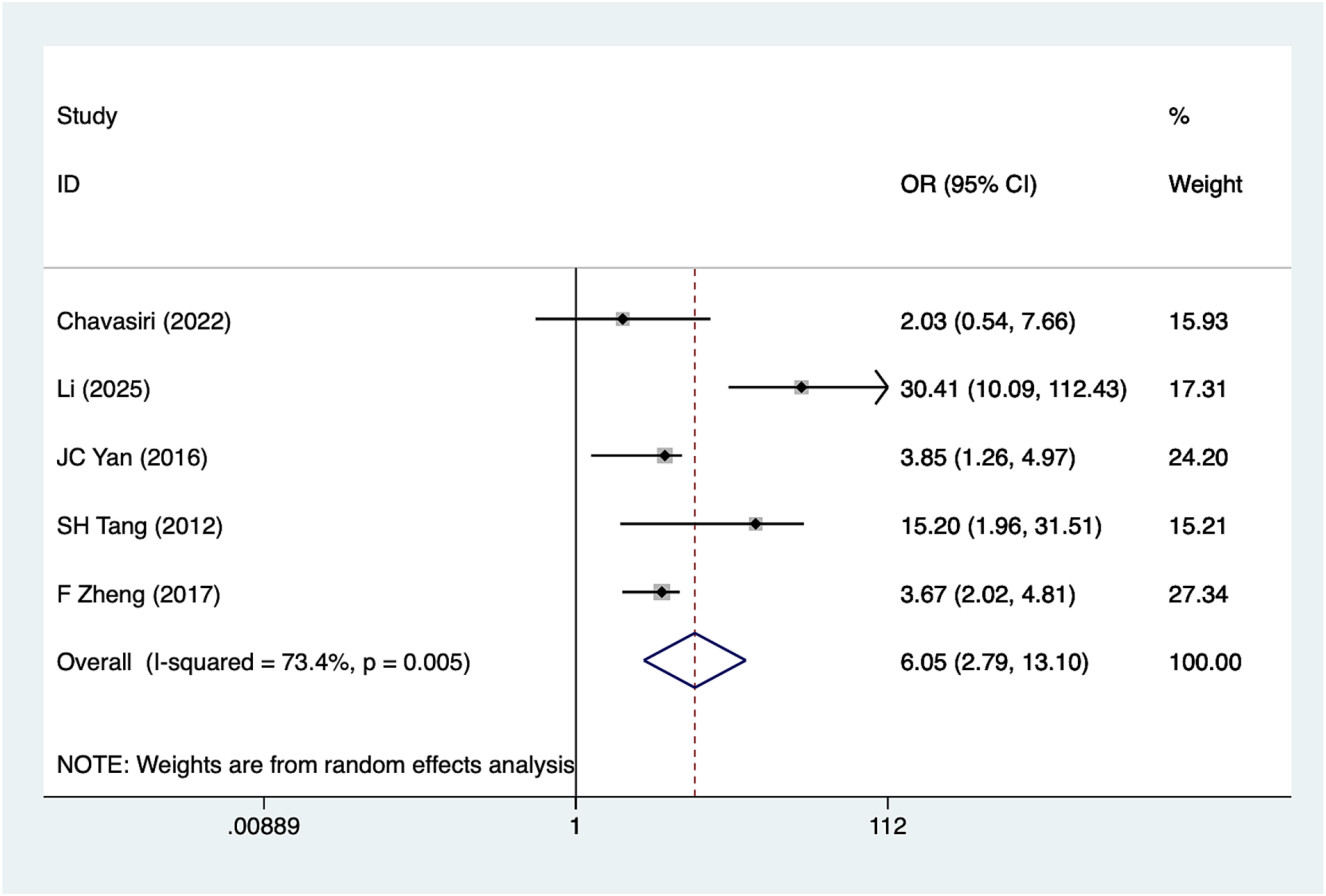
Forest plot of the association between upper cervical spine injury and hyponatremia in patients with acute SCI.

5 studies reported upper cervical spine injury. Heterogeneity analysis (*I*^2^ = 73.4%, *p* = 0.001). Pooled results (Figure 2) indicate an association between upper cervical spine injury and hyponatremia in patients with SCI [OR = 6.05, 95%CI (2.79, 13.10)]. Sensitivity analysis (Supplementary Figure S1) confirms the robustness of this finding, unaffected by any single study. However, this analysis was not sufficient to fully explain the source of heterogeneity.

### Complete SCI

**Figure 3:**
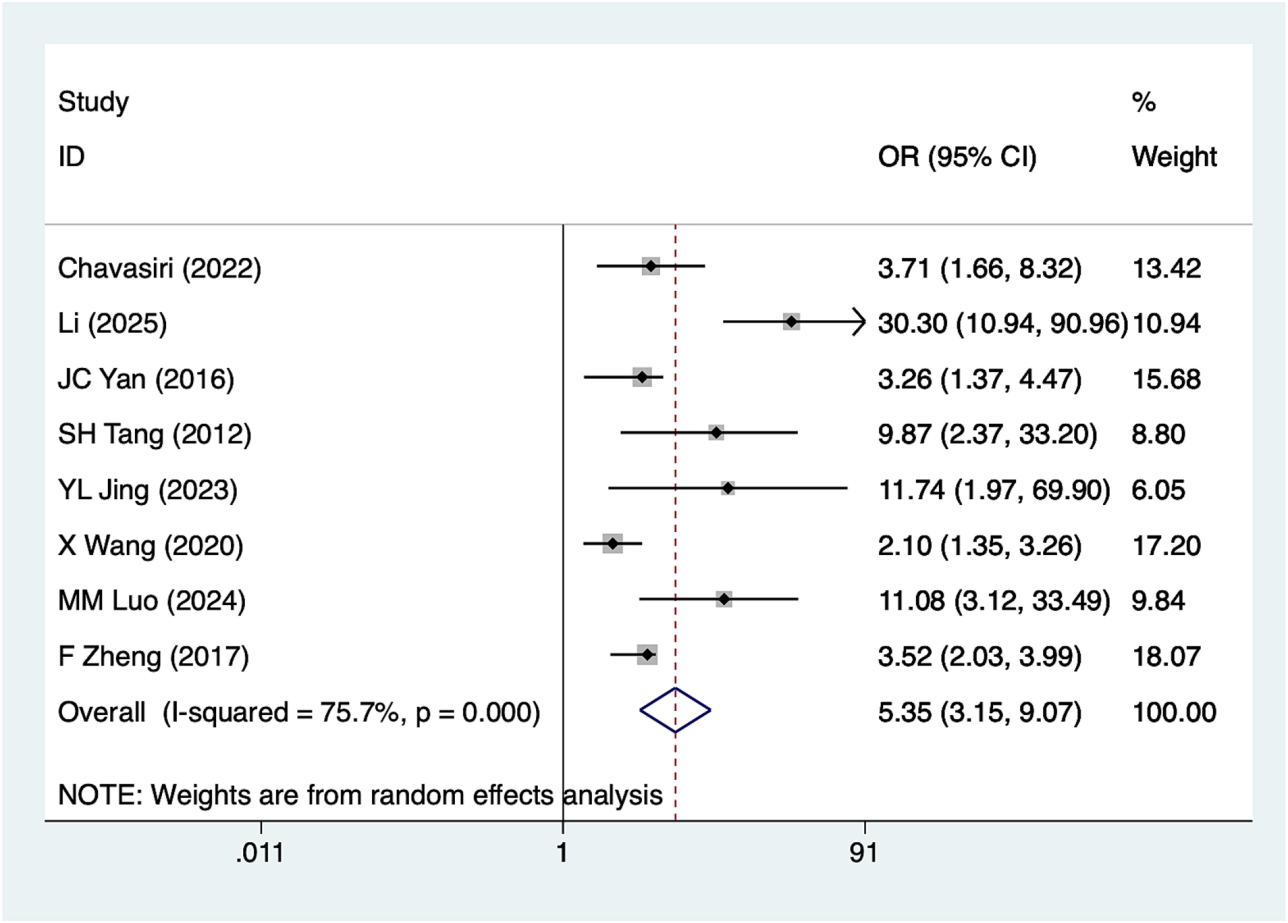
Forest plot of the association between complete SCI and hyponatremia in patients with acute SCI.

Eight studies reported complete SCI. Heterogeneity analysis (*I*^2^ = 75.7%, *p* = 0.001). Pooled results (Figure 3) indicate an association between complete SCI and hyponatremia in patients with SCI [OR = 5.35, 95%CI (3.15, 9.07)]. Sensitivity analysis (Supplementary Figure S2) confirms the robustness of this finding, unaffected by any single study. Nevertheless, the source of heterogeneity could not be fully determined based on sensitivity analysis alone.

### Hypoproteinemia

**Figure 4:**
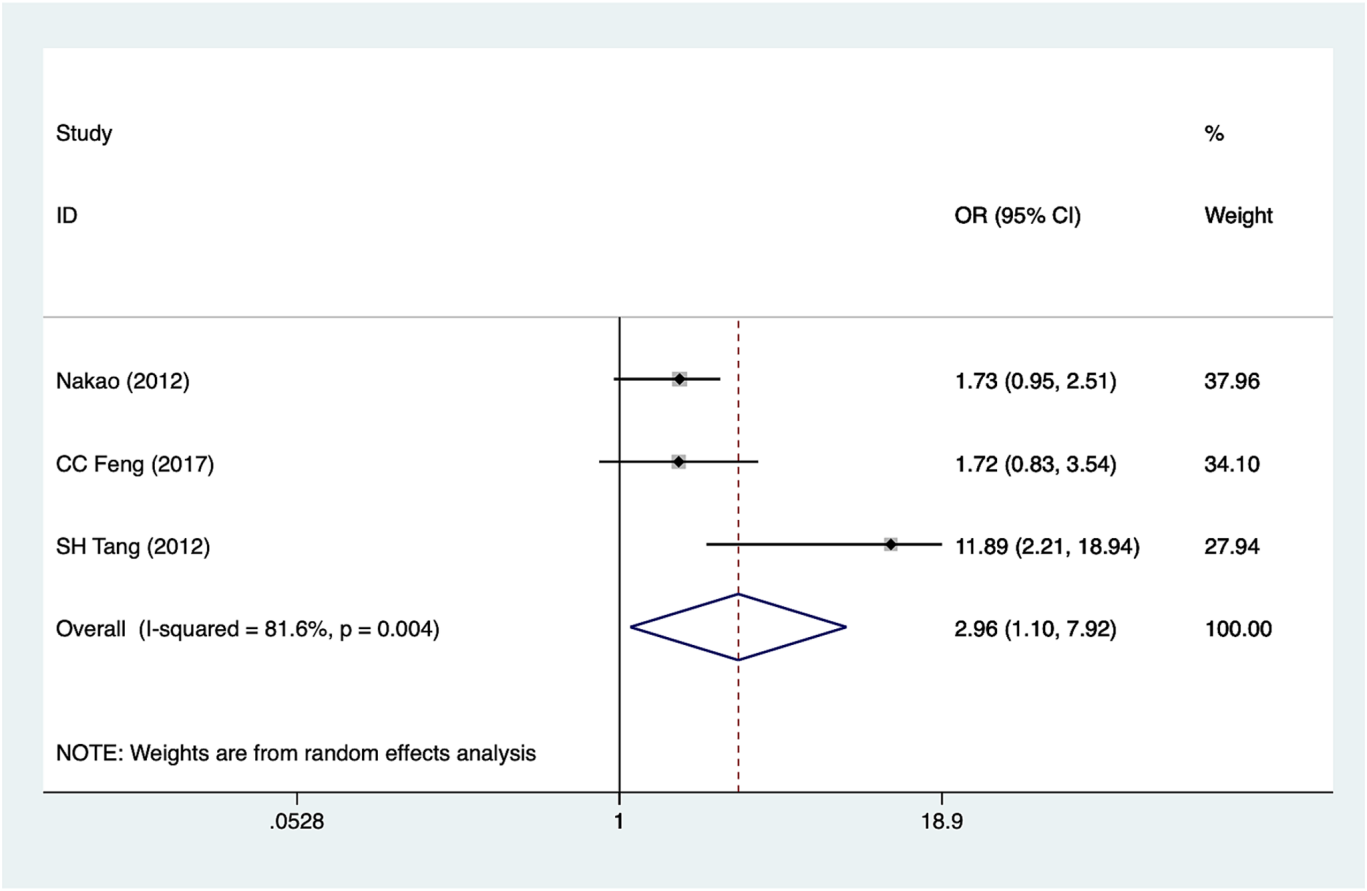
Forest plot of the association between hypoproteinemia and hyponatremia in patients with acute SCI.

Three studies reported hypoproteinemia. Heterogeneity analysis (*I*^2^ = 81.6%, *p* = 0.004). Pooled results (Figure 4) indicate an association between hypoproteinemia and hyponatremia in patients with SCI [OR = 2.96, 95%CI (1.10, 7.92)]. Sensitivity analysis (Supplementary Figure S3) suggested that heterogeneity may originate from Tang (2012) however, given the limited number of included studies, this finding should be interpreted cautiously, and the exact source of heterogeneity remains uncertain.

### Infection

**Figure 5:**
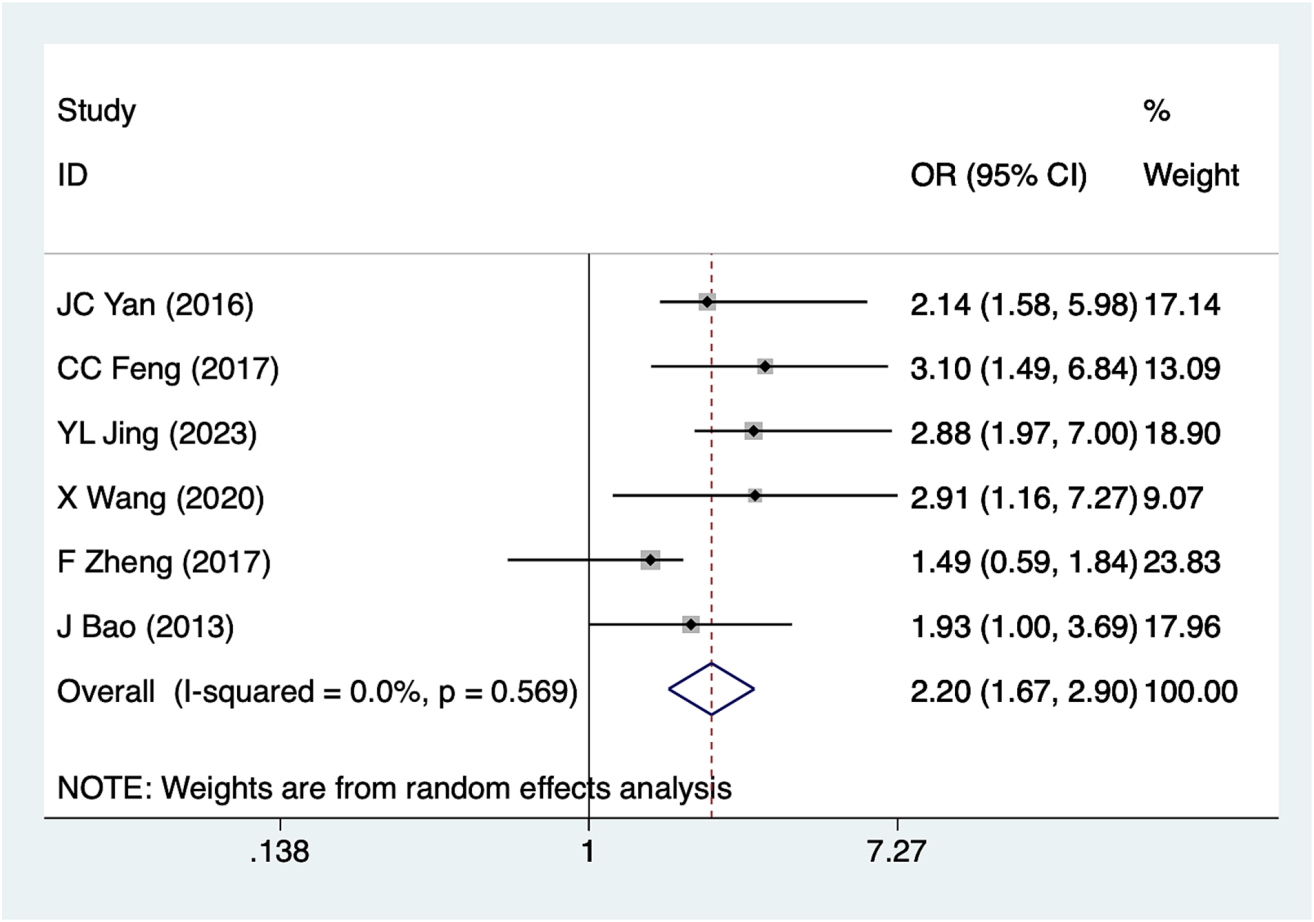
Forest plot of the association between infection and hyponatremia in patients with acute SCI.

Six studies reported infection. Heterogeneity analysis (*I*^2^ = 0%, *p* = 0.569). Pooled results (Figure 5) indicate an association between infection and hyponatremia in patients with SCI [OR = 2.20, 95%CI (1.67, 2.90)].

### Publication bias

This study employed the Egger test and funnel plot analysis to assess publication bias. Results (Supplementary Figures S4–S7) indicate that upper cervical spine injury (Egger *p* = 0.231), hypoproteinemia (Egger *p* = 0.353), and infection (Egger *p* = 0.122) show a low likelihood of publication bias. Complete SCI (Egger *p* = 0.023) showed a higher likelihood of publication bias. Trim-and-fill methods was applied to this outcome (Supplementary Figure S8), and the results indicate that the analysis remains stable even when publication bias is present. However, due to the small number of studies in some categories, conclusions from these findings should be interpreted with caution.

## Discussion

This systematic review and meta-analysis synthesize existing observational evidence to quantitatively assess factors associated with hyponatremia in patients with acute SCI. Results indicate that both high cervical SCI and complete injury are significantly associated with hyponatremia occurrence, exhibiting relatively high effect sizes. Hypoproteinemia and infection are also important contributing factors, with infection demonstrating higher result stability. Overall, this study provides evidence-based support for the critical role of injury level, injury severity, and systemic factors in hyponatremia development, offering further rationale for clinical risk stratification.

Among injury-related factors, high cervical SCI demonstrated the strongest association. High SCI typically causes more severe sympathetic dysregulation, impairing fluid distribution and vascular tone regulation, thereby predisposing to water-sodium imbalance (27, 28). Physiological studies indicate autonomic dysfunction can disrupt antidiuretic hormone secretion regulation, leading to dilutional hyponatremia or salt-wasting changes (29). Additionally, patients with high-level injuries often require intensive respiratory support and fluid therapy, and these medical interventions may also exert cumulative effects on electrolyte homeostasis (30). The findings of this study reinforce the association between injury anatomical level and electrolyte abnormalities from a clinical data integration perspective, suggesting its potential as an important indicator for early risk assessment.

Complete SCI also demonstrated significant associations. Reflecting extensive disruption of neural conduction, complete injury may exert more comprehensive effects on neuroendocrine regulatory networks, thereby amplifying risks of water-salt metabolism imbalance (31). Furthermore, these patients typically present with more severe conditions, longer hospital stays, and higher complication rates—factors that may contribute to a compounding pathway (32). Although this meta-analysis exhibits some heterogeneity, stable sensitivity analysis results indicate consistent overall conclusions (33). However, it should be noted that interaction or collinearity may exist between injury level and severity; future studies should further distinguish their independent contributions using multivariate models.

Regarding modifiable factors, this study observed correlations between hypoproteinemia and hyponatremia. Hypoproteinemia may reflect malnutrition, inflammatory response, or increased disease severity (34). It affects fluid redistribution by lowering plasma colloid osmotic pressure, thereby promoting dilutional hyponatremia (35). However, this finding exhibited high heterogeneity and was significantly influenced by individual studies, suggesting limited evidence that warrants cautious interpretation. Future research should clarify whether hypoproteinemia is an independent risk factor or merely a surrogate marker of disease severity.

Infection showed a relatively stable association with hyponatremia. However, this association should be interpreted cautiously. Infection may contribute to hyponatremia through inflammatory and endocrine pathways, but it may also reflect greater clinical complexity, more intensive treatment exposure, or downstream deterioration during hospitalization (36, 37). Differences in antibiotic use, fluid therapy, ICU care intensity, and supportive management may also have influenced this association. Therefore, infection may be better regarded as a clinical correlate or marker of vulnerability rather than a confirmed independent causal predictor.

Importantly, the associations identified in this meta-analysis should not be interpreted as proof of causality. Because all included studies were observational, the pooled estimates remain susceptible to residual confounding. Some observed associations may have been influenced by differences in treatment and supportive care rather than reflecting true independent biological effects. For example, variability in fluid administration strategies, diuretic exposure, vasopressor use, mechanical ventilation, nutritional support, and ICU monitoring practices may all affect the occurrence or detection of hyponatremia. In addition, patients with more severe injury are more likely to receive intensive care interventions and may also be more likely to develop concurrent complications such as infection or hypoproteinemia, making it difficult to distinguish independent predictors from markers of overall illness severity. Therefore, these findings should be interpreted as clinically relevant associations rather than definitive causal relationships.

From a clinical practice perspective, these findings support risk stratification based on injury characteristics and systemic clinical status. Patients with upper cervical injury or complete SCI may warrant closer serum sodium monitoring, particularly during the early hospitalization period. Likewise, patients with infection or hypoproteinemia may require more careful assessment of fluid balance, nutritional status, and evolving complications. In intensive care settings, electrolyte abnormalities should be interpreted in the context of overall illness severity and supportive care exposure. Although these findings do not establish specific treatment thresholds, they may help clinicians identify patients who require closer surveillance and earlier supportive intervention.

The substantial heterogeneity observed for upper cervical spine injury, complete SCI, and hypoproteinemia should be carefully considered when interpreting these pooled estimates. This heterogeneity may be related to several factors, including differences in study design (cohort versus case–control), patient selection, baseline injury severity, definitions of exposure variables, timing of sodium measurement, clinical management strategies such as fluid administration and intensive care support, and the extent of confounder adjustment across studies. Because only a limited number of studies were available for several risk factors, especially hypoproteinemia, formal subgroup analysis or meta-regression was not feasible with adequate statistical power. Therefore, the sensitivity analyses were used mainly to assess result stability rather than to definitively explain heterogeneity.

### Strengths and limitations

This study has several strengths. It systematically synthesized the available evidence on factors associated with hyponatremia in patients with acute spinal cord injury and provides a focused evidence-based summary of this clinically relevant complication. The use of a consistent diagnostic threshold for hyponatremia across the included studies improved the comparability of outcome definitions. In addition, the application of random-effects models, together with sensitivity analyses and publication bias assessment, strengthened the robustness and interpretability of the pooled findings. By examining both injury-related and systemic clinical variables, this study also offers a broader framework for clinical monitoring and risk stratification.

Several limitations should be considered when interpreting the findings. First, all included studies were observational, and therefore causal inference remains limited. Some variables, particularly infection and hypoproteinemia, may reflect concurrent illness severity or downstream clinical events rather than true independent predictors. Second, substantial heterogeneity was present in several analyses, especially those for upper cervical spine injury, complete SCI, and hypoproteinemia. Although leave-one-out sensitivity analyses supported the relative stability of the pooled estimates, they were insufficient to fully explain the observed heterogeneity. Third, the small number of studies available for some variables reduced statistical power and limited the feasibility of more detailed heterogeneity exploration. Fourth, the adjustment strategies used across studies were not uniform; although adjusted estimates were preferentially extracted when available, differences in the covariates included in multivariable models may have influenced the pooled results. Fifth, most included studies were from China, with only two studies from outside China. This geographic concentration may limit external validity, as the pooled results could be influenced by regional differences in patient characteristics, clinical practice, and healthcare systems. Therefore, caution is needed when extrapolating these findings to other populations. Finally, possible collinearity between injury level and injury severity could not be fully assessed at the study level.

## Conclusion

This study indicates that hyponatremia in patients with acute SCI is associated with multiple clinical factors, with upper cervical spinal cord injury and complete SCI demonstrating stronger correlations. These findings may help identify patients who warrant closer electrolyte monitoring after acute SCI; however, the observed associations should not be interpreted as establishing definite causal relationships, and further prospective studies with adequate confounder control are needed.

Given that most included studies employed observational designs and some outcomes exhibited heterogeneity, conclusions should be interpreted with caution. Future prospective, multicenter, large-sample studies are needed to further validate these associations and explore potential mechanisms, thereby providing higher-quality evidence to optimize clinical management for patients with acute spinal cord injury.
