# Supplementary material for: Factors associated with hyponatremia in patients with acute spinal cord injury: a systematic review and meta-analysis
Source: Front Med (Lausanne). 2026 May 5;13:1829242. doi: 10.3389/fmed.2026.1829242 (PMC13183619; doi:10.3389/fmed.2026.1829242)
Supplement: Supplementary file 1 [file Data_Sheet_1.DOCX]

Table S1 detailed search strategy

(("Spinal Cord Injuries"[Mesh]) OR (((((((((((((((((((((((((((((((((((((((Spinal Cord Injuries[Title/Abstract]) OR (Injuries, Spinal Cord[Title/Abstract])) OR (Cord Injuries, Spinal[Title/Abstract])) OR (Cord Injury, Spinal[Title/Abstract])) OR (Injury, Spinal Cord[Title/Abstract])) OR (Spinal Cord Injury[Title/Abstract])) OR (Myelopathy, Traumatic[Title/Abstract])) OR (Myelopathies, Traumatic[Title/Abstract])) OR (Traumatic Myelopathies[Title/Abstract])) OR (Traumatic Myelopathy[Title/Abstract])) OR (Spinal Cord Trauma[Title/Abstract])) OR (Cord Trauma, Spinal[Title/Abstract])) OR (Cord Traumas, Spinal[Title/Abstract])) OR (Spinal Cord Traumas[Title/Abstract])) OR (Trauma, Spinal Cord[Title/Abstract])) OR (Traumas, Spinal Cord[Title/Abstract])) OR (Post-Traumatic Myelopathy[Title/Abstract])) OR (Myelopathies, Post-Traumatic[Title/Abstract])) OR (Myelopathy, Post-Traumatic[Title/Abstract])) OR (Post-Traumatic Myelopathies[Title/Abstract])) OR (Post Traumatic Myelopathy[Title/Abstract])) OR (Spinal Cord Contusion[Title/Abstract])) OR (Contusion, Spinal Cord[Title/Abstract])) OR (Contusions, Spinal Cord[Title/Abstract])) OR (Cord Contusion, Spinal[Title/Abstract])) OR (Cord Contusions, Spinal[Title/Abstract])) OR (Spinal Cord Contusions[Title/Abstract])) OR (Spinal Cord Laceration[Title/Abstract])) OR (Cord Laceration, Spinal[Title/Abstract])) OR (Cord Lacerations, Spinal[Title/Abstract])) OR (Laceration, Spinal Cord[Title/Abstract])) OR (Lacerations, Spinal Cord[Title/Abstract])) OR (Spinal Cord Lacerations[Title/Abstract])) OR (Spinal Cord Transection[Title/Abstract])) OR (Cord Transection, Spinal[Title/Abstract])) OR (Cord Transections, Spinal[Title/Abstract])) OR (Spinal Cord Transections[Title/Abstract])) OR (Transection, Spinal Cord[Title/Abstract])) OR (Transections, Spinal Cord[Title/Abstract]))) AND (("Hyponatremia"[Mesh]) OR ((Hyponatremia[Title/Abstract]) OR (Hyponatremias[Title/Abstract])))


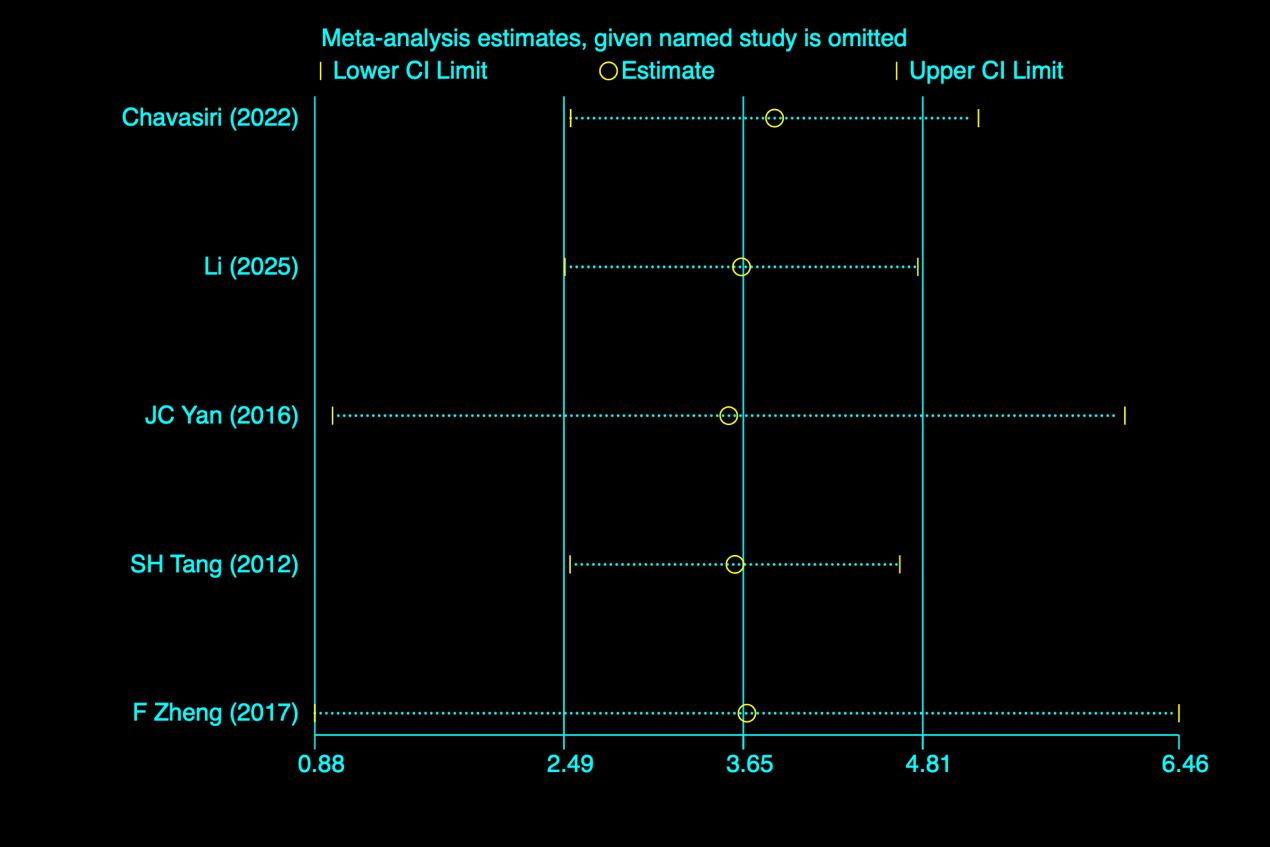


Figure S1 Sensitivity analysis of Upper cervical spine injury


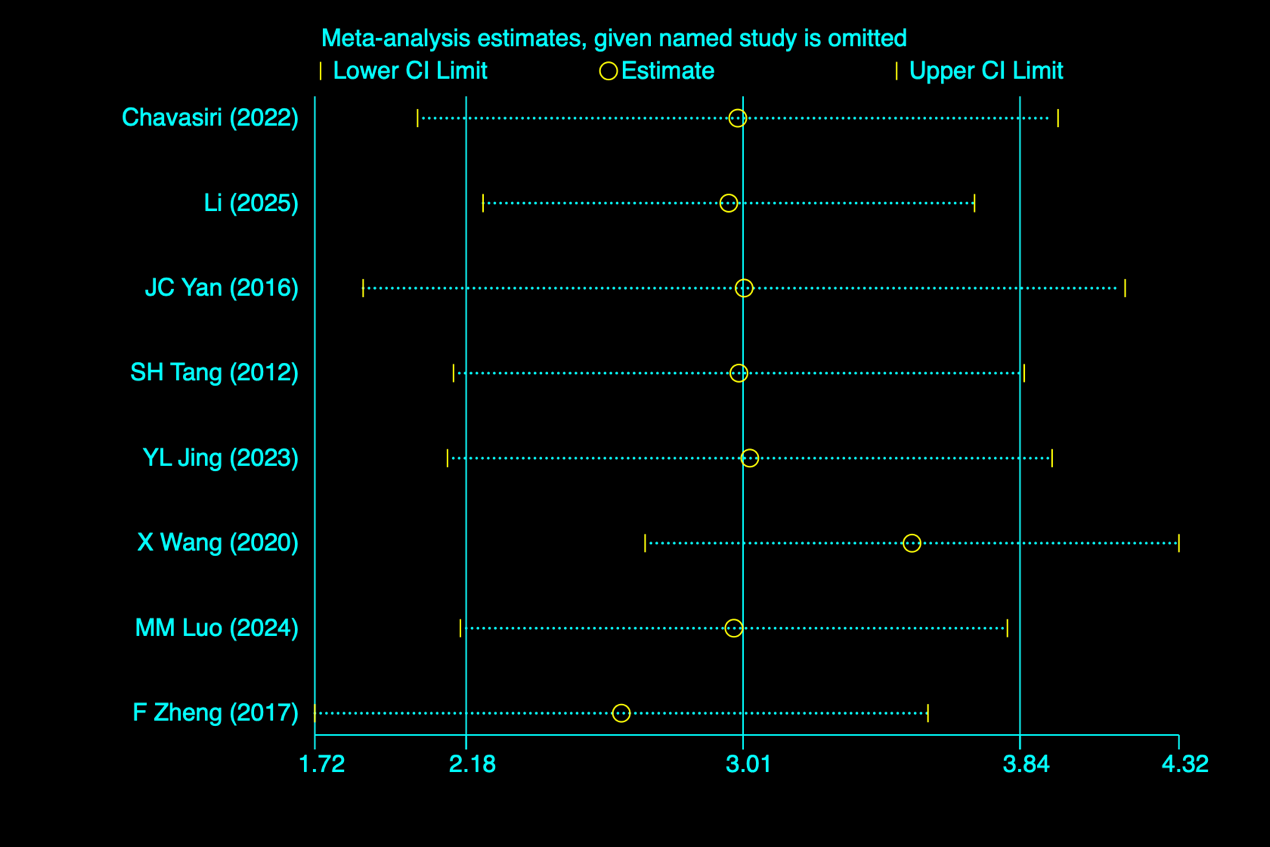


Figure S2 Sensitivity analysis of Complete SCI


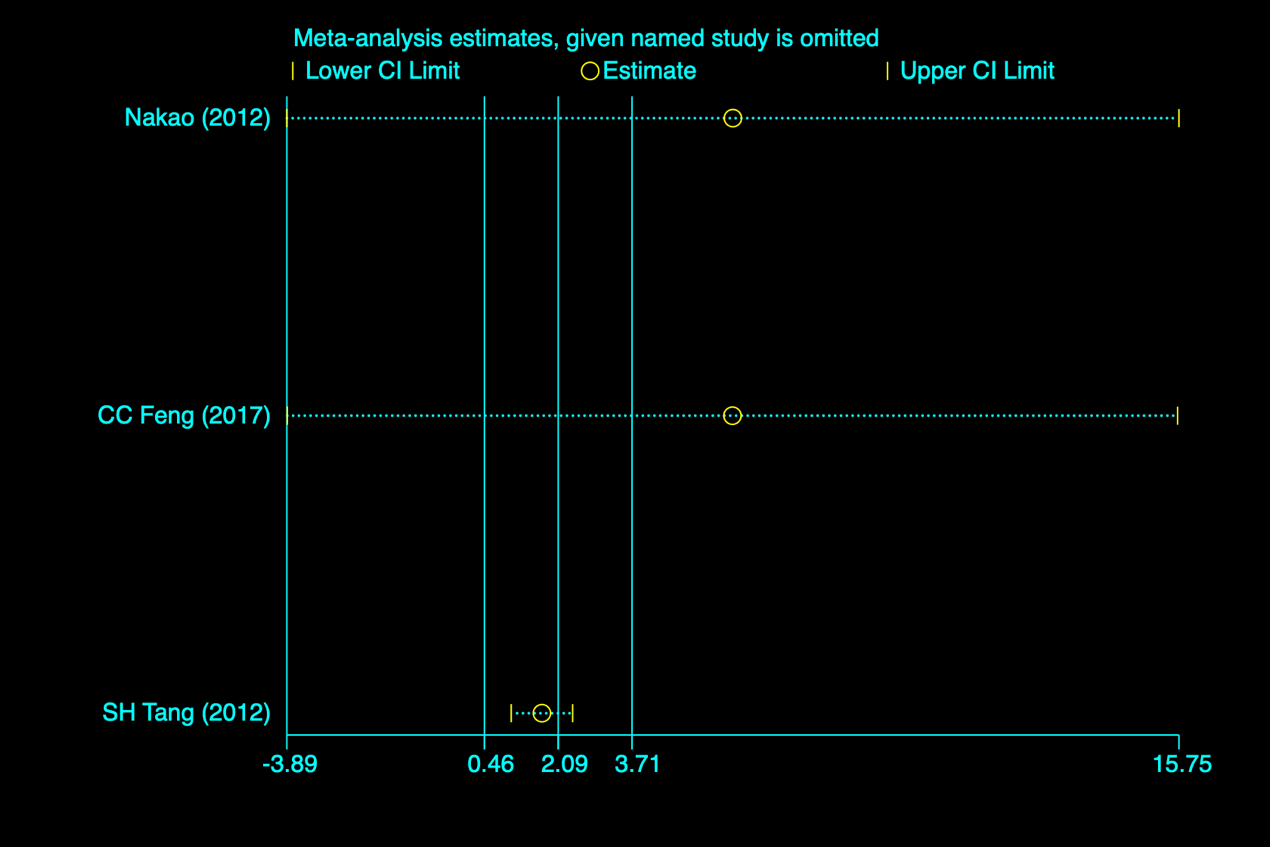


Figure S3 Sensitivity analysis of hypoproteinemia


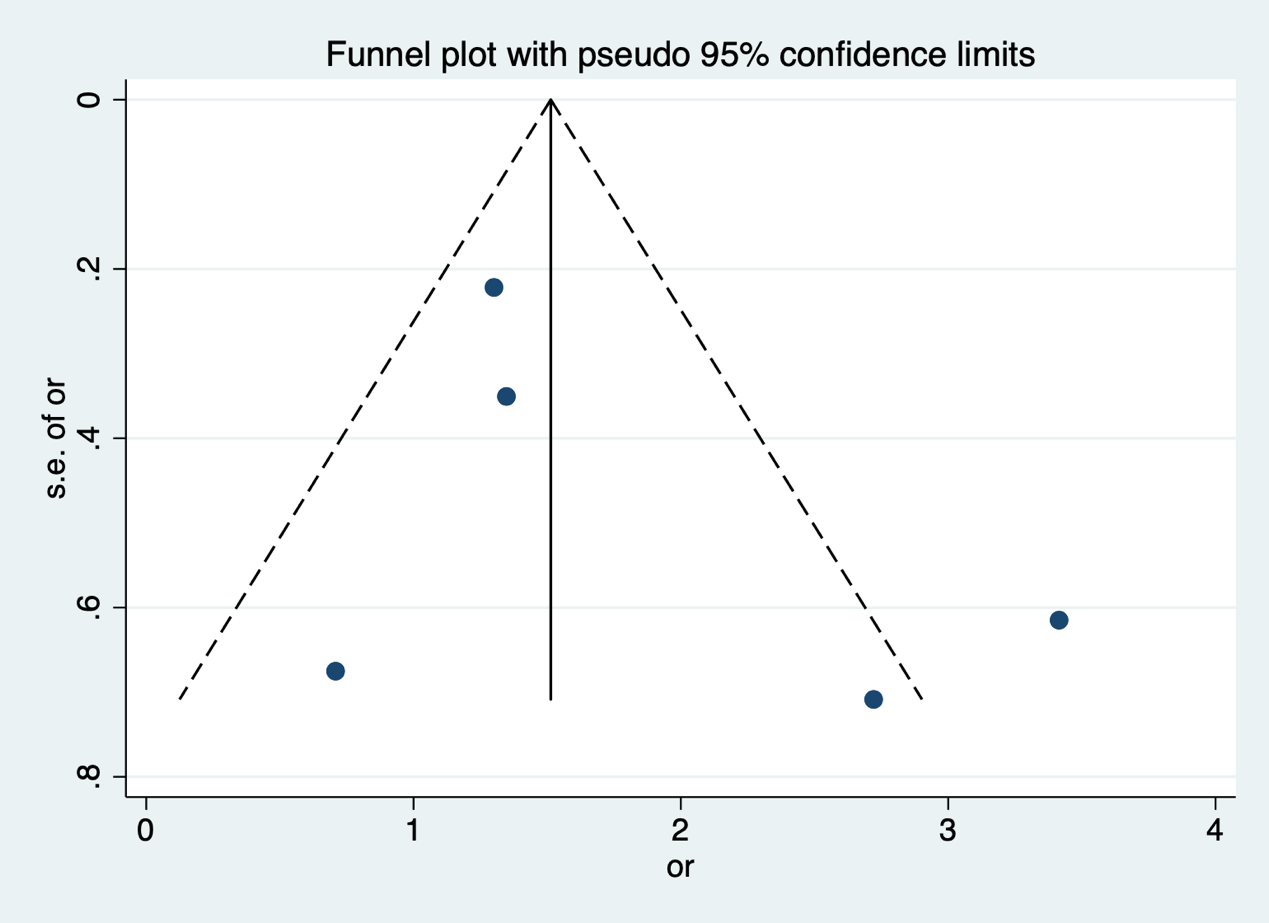


Figure S4 Funnel plot of meta-analysis of Upper cervical spine injury


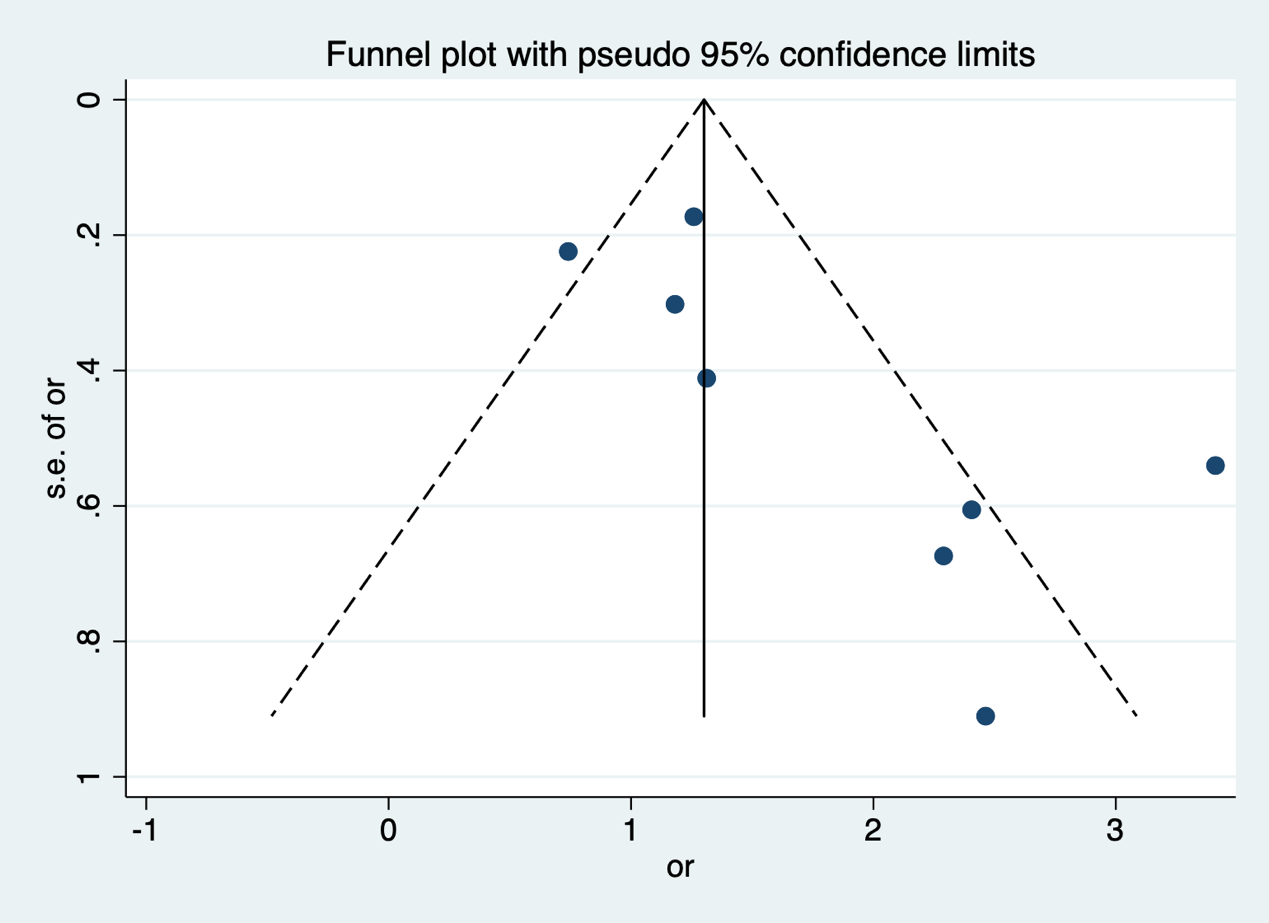


Figure S5 Funnel plot of meta-analysis of Complete SCI


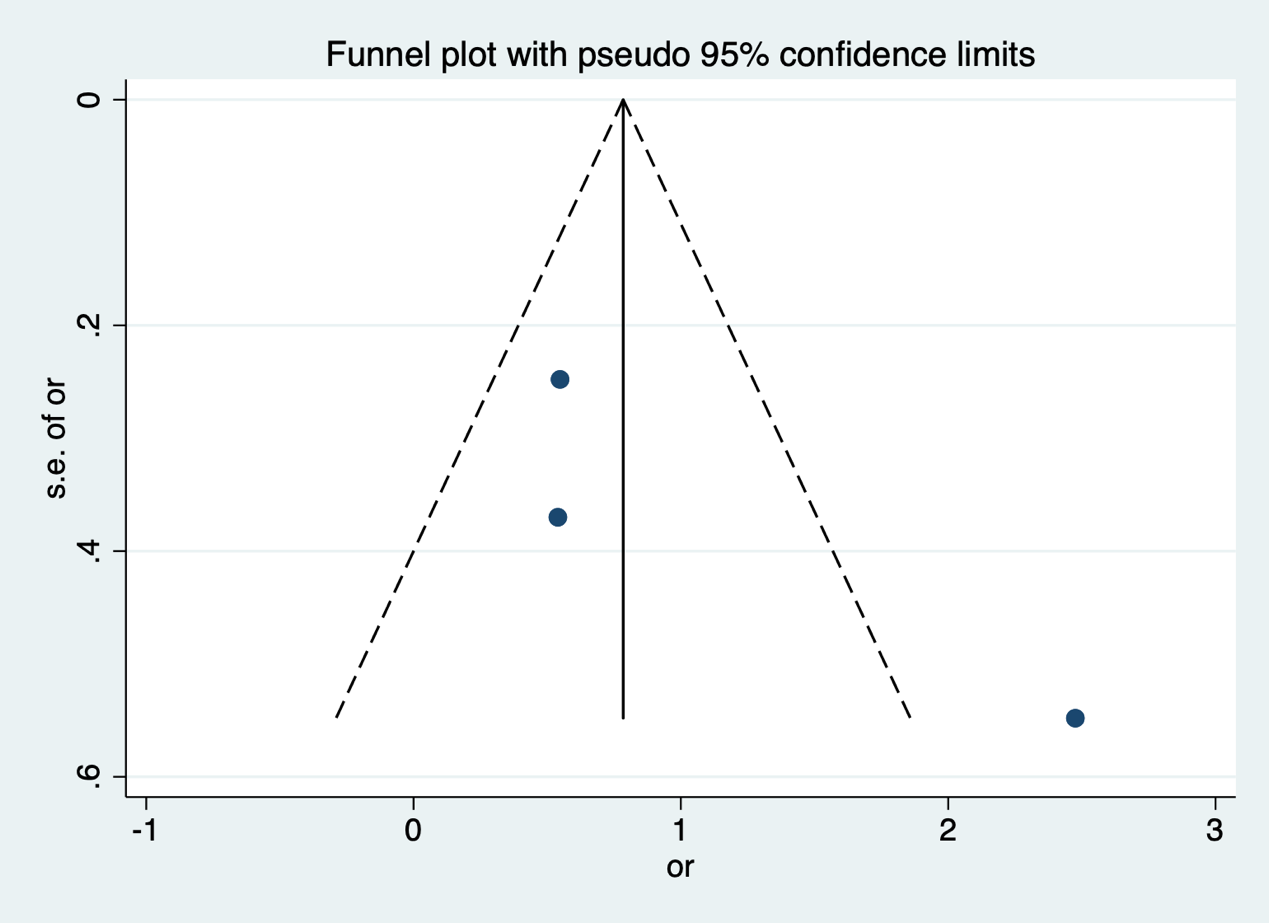


Figure S6 Funnel plot of meta-analysis of hypoproteinemia


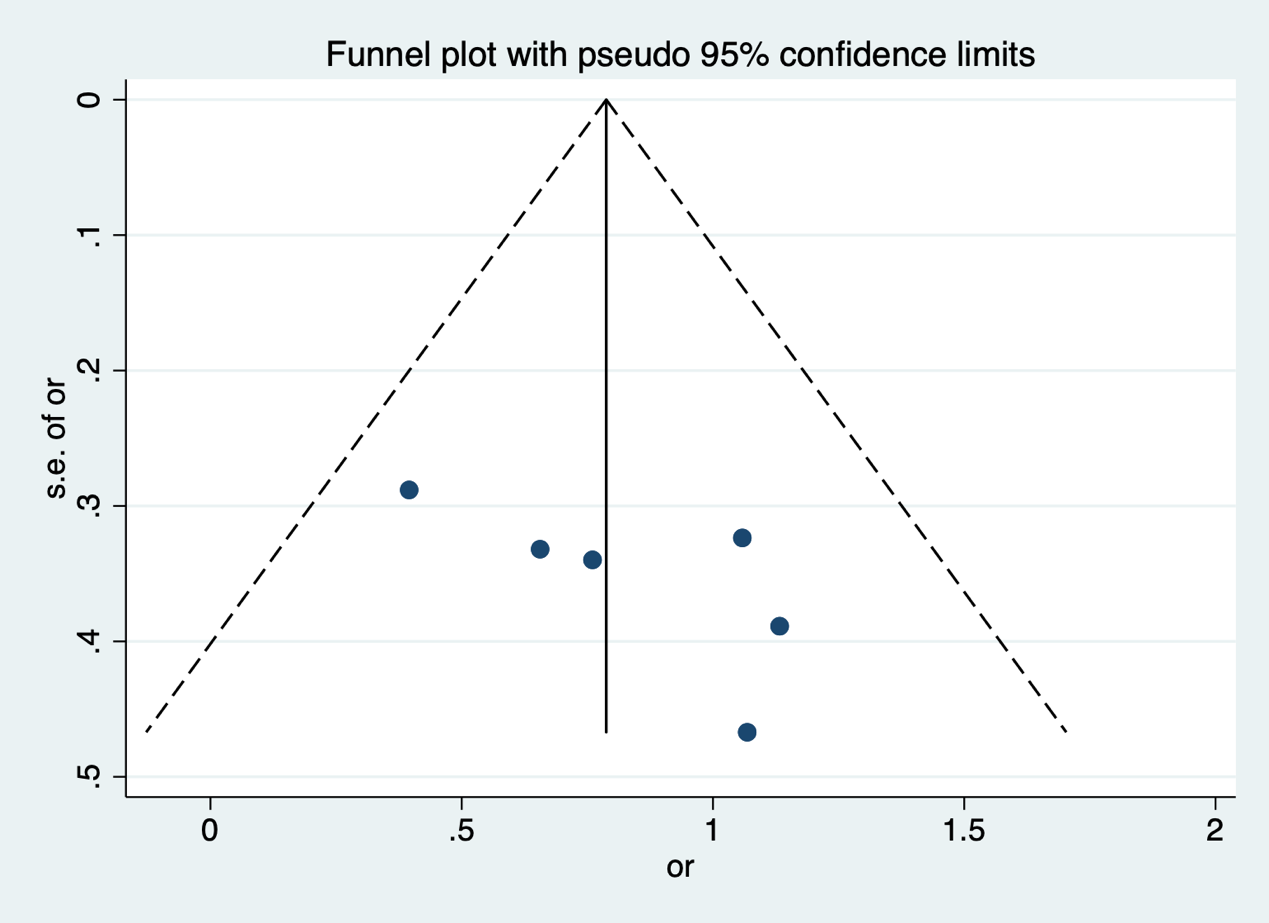


Figure S7 Funnel plot of meta-analysis of infection


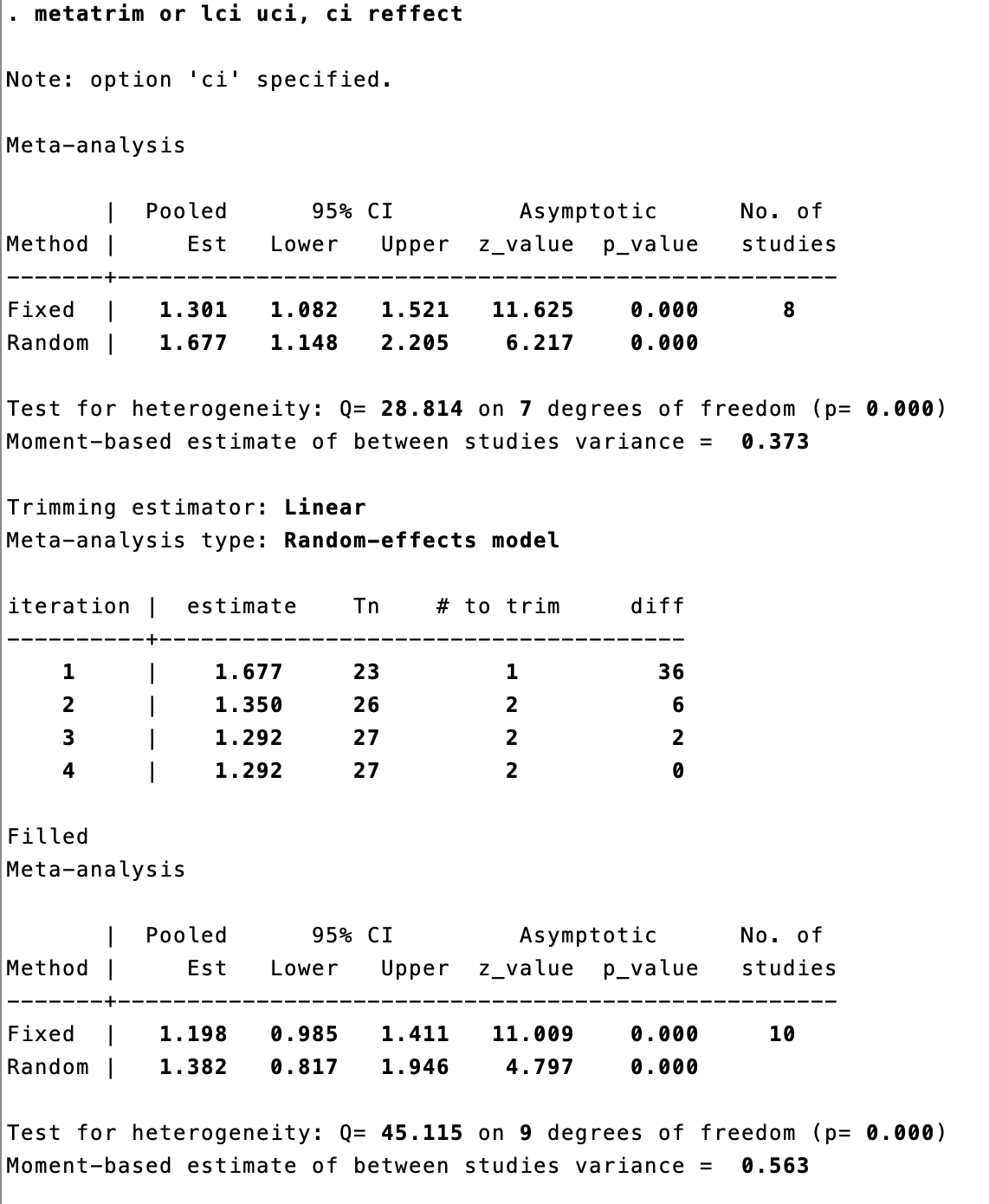


Figure S8 Results of the trim-and-fill for complete SCI
